# Gastroparesis associated with gastroptosis presenting as a lower abdominal bulking mass in a child: a case report

**DOI:** 10.1186/1757-1626-2-184

**Published:** 2009-11-04

**Authors:** Efstratios Christianakis, Konstantina Bouchra, Aikaterini Koliatou, Nikolaos Paschalidis, Dimitrios Filippou

**Affiliations:** 1 Departments of Pediatric Surgery, Pendeli Children's General Hospital, Athens, Greece; 2 Departments of Radiology, Pendeli Children's General Hospital, Athens, Greece; 3 Department of Anatomy and Embryology, Nursing Faculty, University of Athens, Greece

## Abstract

**Background:**

Gastroparesis is defined as an inhibition of the gastric motility associated with delayed gastric emptying, which is mainly presented with acute dyspepsia. Gastroptosis is the downward displacement of the stomach.

**Case Report:**

We report a rare case of secondary gastroparesis due to gastroptosis in an 11-year-old female child. The patient complained for bulking mass in the left lower quadrate presented a week ago, which was mimicking a large abdominal hernia. The laboratory and radiological exams revealed an excessive gastroptosis associated with gastroparesis. We searched the literature but we failed to find other cases with idiopathic gastroptosis in a child. The patient was treated conservatively and six months after the initial diagnosis and treatment the patient does not complains for dyspepsia.

**Conclusion:**

Gastroparesis associated with gastroptosis is a rare entity that can be treated conservatively with acceptable results.

## Background

Gastroptosis is defined as the downward displacement of the stomach [1]. Since nowadays only few sporadic cases of this rare entity have been reported in the literature and all of them refer to adult patients [1-5]. Gastroparesis is inhibition of the gastric motility which characterized as paralysis, and is also very rare in children [1]. A detailed of the literature by using the relative terms failed to find similar reports of both these entities co-existing in children. We are presenting a unique case of gastroparesis associated with gastroptosis in a female child presented as painful bulking lower abdominal mass.

## Case Presentation

**Figure 1:**
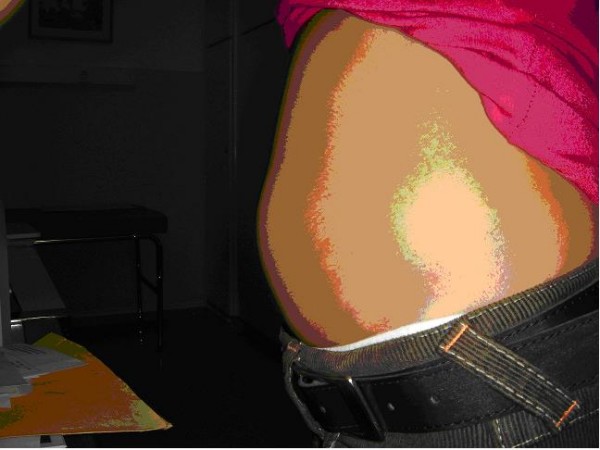
**Subumbilical painful abdominal bulge, after running**.

An 11-year-old female patient proceeded in the Emergencies Department of our hospital complaining for a painful abdominal bulking mass that has been presented seven days ago. The clinical examination revealed a large painful bulking mass in the left lower abdomen, which was disappearing in supine position (figure 1). We did not manage to palpate a deficit or split in the abdominal wall.

The parents characterized their child as a neurotic child suffering from dyspeptic symptoms for more than two years that had been defined and treated as gastritis by per os administration of ranitidine and magnesium hydroxide. Even the medical treatment was still vomiting, while she had breakfast with milk and during traveling. Furthermore she avoided eating large amounts of food, because of gastric pain and hooves. All blood tests that were performed were normal (Glucose 95 mg/dl, Urine 30 mg/dl, Creatinine 0,6 mg/dl, k 3,9 mEq/l, Na 142 mEq/l, Cl 105 mEq/l, T3 1,4 ng/ml, T4 8 mg/dl, TSH 0,6 UI/ml).

**Figure 2:**
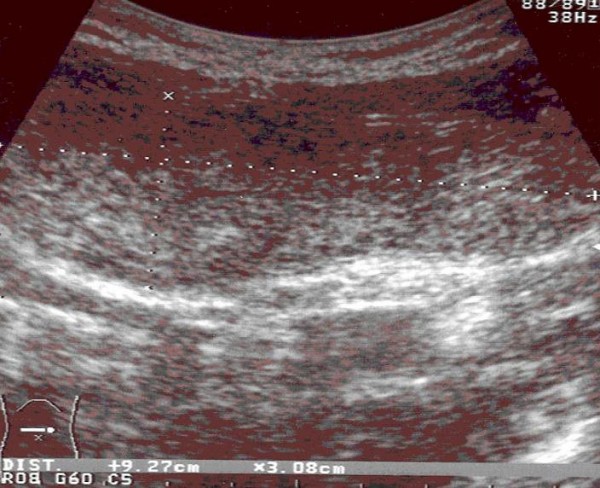
**Longitudinal ultrasonogram of the abdomen showing continuity of the stomach and its contents in the periumbilical region**.

**Figure 3:**
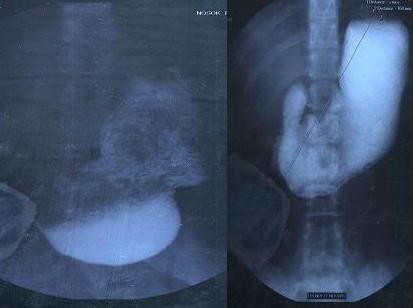
**Barium study examination in erect position, showing elongation of the stomach down to the level of the iliac crests**.

We performed abdominal ultrasonography (figure 2) and barium studies (figure 3) that revealed a dilated stomach, in which the contrast medium was identified lower than the iliac crest in the left iliac fossa. The abdominal MRI didn't reveal any parietal or aponeurotic deficit. Upper GI endoscopy showed an elongated gastric utricle and evidence of reduced clearance of gastric contents (semi-solid meal and bile staining liquids) as well as a mild gastritis of the andrum. The histological examination of multiple biopsies received was negative for H. Pylori infection or any other mucosal pathology.

Radio isotopic study of gastric evacuation with solid meal revealed a significant distention of the stomach along with prolonged halftime in the evacuation time of a solid meal.

The above-mentioned data suggest the diagnosis of gastroparesis with simultaneous gastroptosis. The patient was treated medically with per os administration of *domperidon*. The initial dose of *Domperidon *was 10 ml, four times per days for three months, followed by a dose of 10 ml three times per day. Six months after the initial diagnosis and treatment the bulking mass has been disappeared and the patient does not complain for pain, dyspepsia or discomfort.

## Discussion

**Gastroparesis **is a disorder of gastric motility which is mainly characterized by epigastric pain, postprandial fullness, anorexia, satiety, bloating, vomiting and halitosis [2]. In patients with gastroparesis the food absorption is delayed and unpredictable. Gastroparesis may occur in cases of disfunction or trauma of the gastric nervous system. In cases of vagus nerves damage, the GI musles may function abnormally affecting the downward movement of the food [3].

The diagnosis of gastroparesis can be achieved by barium studies, radioisotopic food or liquid gastric empting studies, androduodenal manometry, sonography, (13) C-acetate breath test and evaluation of gastric electrical activity [2,4-6]. In a cohort of 11 children with persistent gastroparesis after an acute viral illness (eight with rotavirus infection) gastric delayed emptying had been demonstrated scintigraphically in 10 of them. Antroduodenal manometry confirmed postprandial antral hypo motility in 10 of them. The authors refer that all children recovered within 6 to 24 months [7].

The aetiology of gastroparesis in children differs from this in adults [8]. In adults, in most of the cases gastroparesis is either idiopathic or associated with diabetes mellitus. The most common causes of gastroparesis include diabetes mellitus (type 1 or 2), gallbladder diseases, pancreatitis, post viral syndromes, anorexia nervosa, gastric damage or surgery, vagus nerve damage, medications, gastroesophageal reflux disease, gastric cancer, smooth muscle disorders, amyloidosis, scleroderma, abdominal migraine, Parkinson's disease, metabolic disorders and hypothyroidism. During childhood, gastroparesis is quite rare, and is mainly occurs in preterm infants, with either immaturity of the gastrointestinal tract, or when there is allergy to cow's milk protein. Delayed gastric emptying may be associated with diabetes mellitus, viral infections. hypothyroidism, eosinophilic gastroenteropathy, muscular dystrophy or vagotomy [8]. Delayed gastric emptying time and gastric electrical signal abnormalities are not an unusual finding in children with diabetes mellitus. These patients should be investigated for abnormalities in gastric motility [3].

In most of the cases occur in adults, gastroparesis cannot be managed with any kind of treatment, and usually becomes a chronic subclinical problem. Various therapeutic strategies have been proposed including insulin administration, oral medications, changes in eating habits, and in severe cases enteral and parenteral nutrition. Therapeutic approaches in children differ from those in adults. The therapeutic strategy in children is focused on restoration of the nutritional status, as well as in administration of antivomiting medication and medication to improve gastric motility. Domperidone, metoclopramide, erythromycin, cisapride and tegaserod have been shown that may improve gastric emptying [2]. Gastric electrical stimulation seems a promising alternative therapeutic option, which may be available at present [8,9]. Many other therapies as botuline toxin injection into the pyloric muscle and pyloric dilatation may help by reducing the gastric outlet pressure [10]. Some author authors suggest surgical treatment with decompressive gastrostomy, pyloroplasty, pyloromyotomy or feeding jejunoplasty [9,11]. Most babies will outgrow their delayed gastric emptying problems as their digestive track matures and becomes more coordinated.

**Gastroptosis **is the abnormal downward displacement of the stomach. Although this condition is not life threatening is associated with constipation, discomfort, vomiting, dyspepsia, tenesmus, anorexia, nausea and belching. An after-meal activity in patients with gastroptosis may induce nausea and discomfort. Saggital downward displacement of stomach is often caused by relaxation, stretching, or decrease of the muscles tone and may associated with delaying in digestion and in GI tract distension with gas that induces constipation. Gastroptosis may also associate with incorrect posture. Gastroptosis is more a pathological condition than a disease and is classified as hereditary and acquired [12].

In the beginning of 19^th ^century the preferred treatment was surgical [13-15]. In 1894, Duret first reported surgical treatment of gastroptosis in a patient in Lille. Nowadays surgical treatment is still an alternative approach in the treatment of gastroptosis although most authors suggest that should be limited to selected cases [7,16].

Other therapeutic modalities that have been proposed for the treatment of this entity with acceptable results and safety include acupuncture and herbal treatment [17].

Gastroparesis and gastroptosis can be easily misdiagnosed due to their rarity. In our case the patient was treated for gastritis for two years, without having examined by barium studies. *Domperidone *had been proved to be effective in the treatment of gastroparesis by reducing dyspepsia usually within six months.

## Conclusion

In conclusion, persisting dyspepsia in children, particularly in females, should be thoroughly examined for the possibility of gastroparesis. Delayed diagnosis or misdiagnose of gastroparesis may lead secondarily to gastroptosis.

## Competing interests

The authors declare that they have no competing interests.

## Authors' contributions

EC participated in the patients treatment, had the concept of the case report and contributed in the first draft, NP participated in the first draft and in the revisions, KB and EK participated in the diagnosis of the case and in the presentation of its pathology, and DF participated in patient's treatment, co-wrote the first draft and performed all the revisions. All authors read and approved the final manuscript.

## Consent

Written informed consent was obtained from the patient for publication of this case report and accompanying images. A copy of the written consent is available for review by the Editor-in-Chief of this journal.
